# Supplementary material for: Sleep-Wake Transitions Are Impaired in the AppNL-G-F Mouse Model of Early Onset Alzheimer’s Disease
Source: bioRxiv. 2025 Dec 29:2025.12.29.696818. Preprint. [Version 1] doi: 10.64898/2025.12.29.696818 (PMC12776331; doi:10.64898/2025.12.29.696818)
Supplement: Supplement 1 [file NIHPP2025.12.29.696818v1-supplement-1.pdf]

bioRxiv preprint doi: https://doi.org/10.64898/2025.12.29.696818; this version posted December 29, 2025. The copyright holder for this preprint (which was not certified by peer review) is the author/funder, who has granted bioRxiv a license to display the preprint in perpetuity. It is made available under aCC-BY 4.0 International license.

Supplementary Figure Legends

Figure S1. Multiple Sleep Latency Test (MSLT) results from 18-24 month old *App*$^{WT/WT}$ (WT) and *App*$^{NL\text{-}G\text{-}F/NL\text{-}G\text{-}F}$ (KI) mice. **A**. Percent time in NREM sleep during each 20 min nap opportunity in WT (black) and KI (red) mice. The five 20 min SD periods are indicated by horizontal bars below the abscissa. **B.** Percent time in REM sleep during each 20 min nap opportunity. **C.** Mean percent NREM time during the 5 nap opportunities in WT and KI mice. **D.** Mean NREM sleep latency in the WT and KI mice. **E**. Mean percent REM time during the 5 nap opportunities in WT and KI mice. **F**. Mean REM sleep latency between WT and KI mice. Values are mean ± SEM. **, $p < 0.01$.

Figure S2. Arousal state amounts during baseline (BL) and during recovery sleep (RS) during the 6-h of the light phase after cessation of 6-h sleep deprivation (left) and the subsequent 12-h dark phase (right) in *App*$^{WT/WT}$ (WT) and *App*$^{NL\text{-}G\text{-}F/NL\text{-}G\text{-}F}$ (KI) mice. **A** and **A'**. Mean hourly amounts of Wakefulness. **B** and **B'**. Mean hourly amounts of NREM sleep. **C** and **C'.** Mean hourly amounts of REM sleep. Values are mean ± SEM. *, $p < 0.05$; **, $p < 0.01$; ***, $p < 0.001$; ****, $p < 0.0001$.

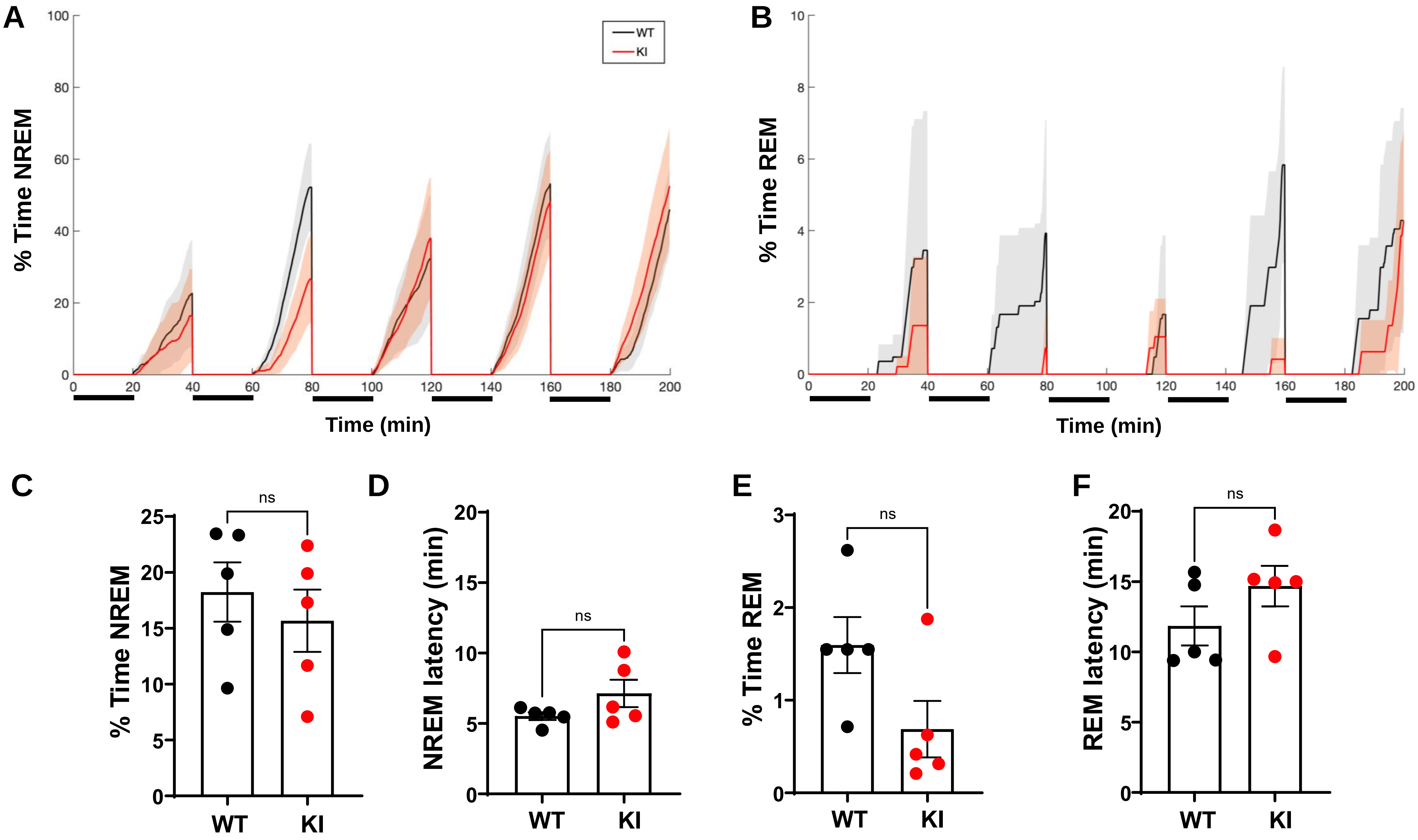
A
% Time NREM
Time (min)
WT
KI
B
% Time REM
Time (min)
C
% Time NREM
ns
WT
KI
D
NREM latency (min)
ns
WT
KI
E
% Time REM
ns
WT
KI
F
REM latency (min)
ns
WT
KI

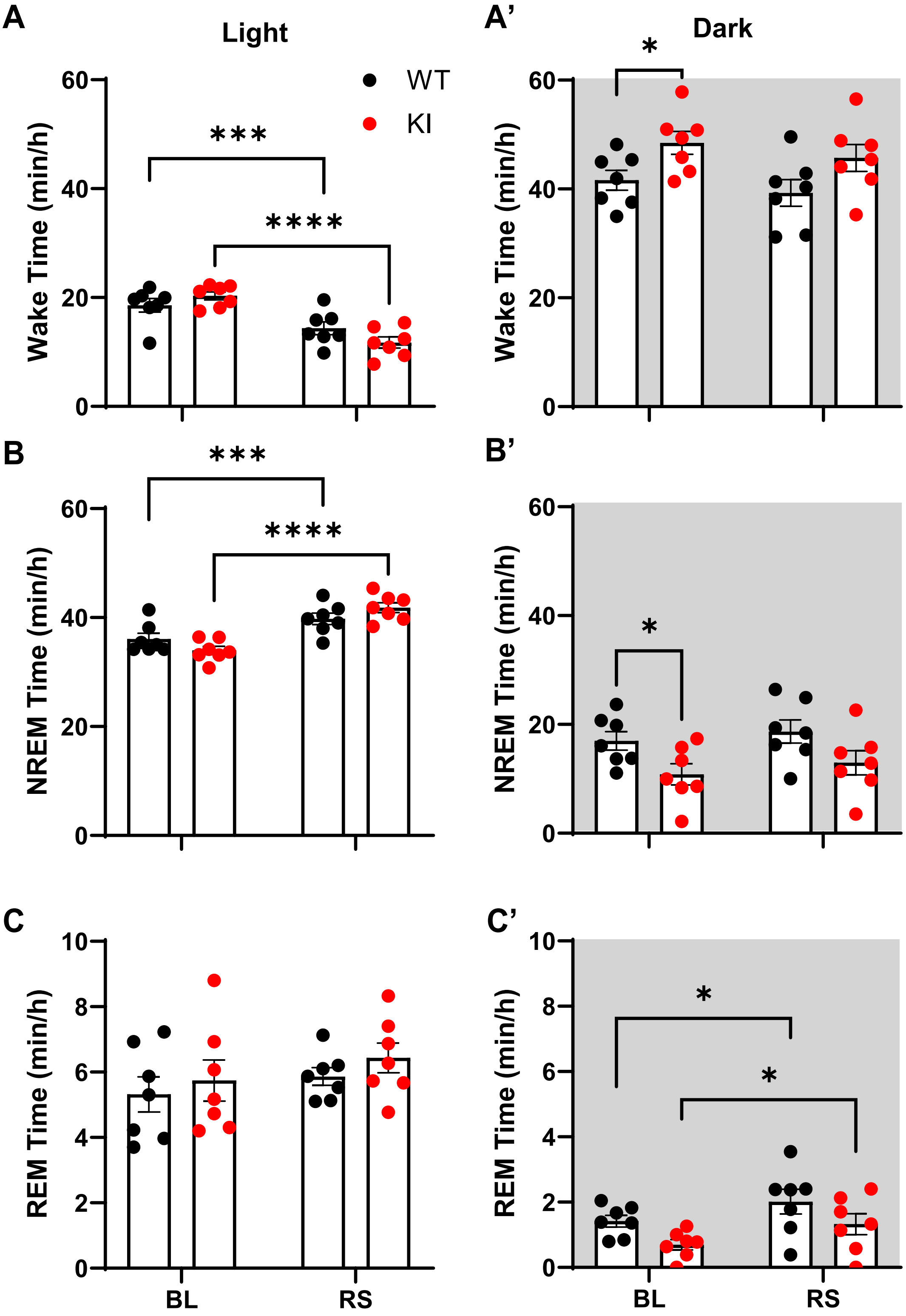
A
Light
A'
Dark
WT
KI
***
****
*
Wake Time (min/h)
B
B'
NREM Time (min/h)
C
C'
REM Time (min/h)
BL
RS
0
20
40
60
0
2
4
6
8
10
